# Conditioned medium from BV2 microglial cells having polyleucine specifically alters startle response in mice

**DOI:** 10.1038/s41598-022-23571-5

**Published:** 2022-11-04

**Authors:** Ryuji Owada, Yohei Kakuta, Kosuke Yoshida, Shinichi Mitsui, Kazuhiro Nakamura

**Affiliations:** 1 Department of Laboratory Sciences, Gunma University Graduate School of Health Sciences, 3-39-22, Showa-Machi, Maebashi, Gunma 371-8511 Japan; 2 Department of Orthopedic Surgery, Gunma University Graduate School of Medicine, 3-39-22, Showa-Machi, Maebashi, Gunma 371-8511 Japan; 3 Department of Rehabilitation Sciences, Gunma University Graduate School of Health Sciences, 3-39-22, Showa-Machi, Maebashi, Gunma 371-8511 Japan

**Keywords:** Diseases of the nervous system, Neurological disorders

## Abstract

Repeat-associated non-AUG translation (RAN translation) is observed in transcripts that are causative for polyglutamine (polyQ) diseases and generates proteins with mono amino acid tracts such as polyalanine (polyA), polyleucine (polyL) and polyserine (polyS) in neurons, astrocytes and microglia. We have previously shown that microglia with aggregated polyQ led to defective differentiation and degeneration of neuron-like cells. However, it has not been determined whether only microglia containing a specific RAN product, but not other RAN products, is harmful in vitro and in vivo. Here we show that polyL-incorporating microglia specifically led to altered startle response in mice. Aggregated polyA, polyS and polyL induced aberrant differentiation of microglia-like BV2 cells. Differentiated PC12 cells treated with conditioned medium (CM) of polyS- and polyL- but not polyA-incorporating microglia-like BV2 cells showed retraction of neurites and loss of branch of neurites. Injection of the polyL-CM, but not polyA-CM and polyS-CM, into the lateral ventricle lowered startle response in mice. Consistently, polyL induced the highest expression of CD68 in BV2 cells. The lowered startle response was replicated in mice given the polyL-CM in the caudal pontine reticular nucleus (PnC), the key region of startle response. Thus, endogenous RAN proteins having polyL derived from polyQ diseases-causative genes in microglia might specifically impair startle response.

## Introduction

Polyglutamine (polyQ) diseases include multiple neurodegenerative disorders such as dentatorubropallidoluysian atrophy, spinal and bulbar muscular atrophy, several types of spinocerebellar ataxia (SCA) and Huntington’s disease (HD)^1^. The region that is responsible for the pathogenesis is polyQ tract in the causative protein of each polyQ disease. When the polyQ repeats are expanded above the thresholds, the symptoms are observed^2^. The extended polyQ repeats are brought by hereditarily expanded CAG repeats in the corresponding causative genes. Neuronal dysfunctions and cell death in polyQ diseases can be mainly explained in a cell-autonomous fashion. The extended polyQ in neurons is prone to form oligomers and fibrils in the cytoplasm and the nucleus where these aggregates exert toxicity to neurons^2–14^. However, neural impairments in a non cell-autonomous fashion have been also proposed in polyQ diseases based on some observations.

Microglia plays crucial roles in physiological brain functions via phagocytosis and scanning of the CNS^15,16^. However, microglia can also give toxic effects to the brain^16^. For instance, neurons in mice having mutant Huntingtin (HTT), the causative protein of HD, only in microglia had high cell death rate under sterile inflammation condition^3^. Likewise, cultured microglial cells having aggregated peptide with 69 glutamine repeat (69Q) showed morphological changes and its conditioned medium (CM) induced degeneration of neuron-like cells^17^. The non cell-autonomous effects by microglia having mutant polyQ might be associated with polyQ diseases because nuclear mutant HTT inclusions were detected in microglial cells in the frontal cortex of adult-onset HD and in the frontal cortex and striatum of juvenile-onset HD^18^.

Recently, repeat-associated non-AUG (RAN) translation from polyQ diseases-causative genes with CAG repeats has been reported^19^. Upon the RAN translation, proteins containing polyalanine (polyA), polyserine (polyS), polyleucine (polyL) and polycystein (polyC) can be theoretically generated in addition to polyQ. PolyQ and polyA RAN proteins were generated from an ataxin 3 gene, a causative gene for SCA type 3^20^. Similarly, polyA-, polyS-, polyL- and polyC-containing proteins were detected in the brain from patients with HD^21^. The RAN proteins are likely toxic to the brain because injection of polyS and polyL aggregates into the lateral ventricle led to altered brain functions in mice^22^.

Notably, the RAN proteins were found in neurons, astrocytes and microglia in the caudate and putamen from patients with HD^21^. Although contributions of polyQ- incorporating microglia to neural dysfunctions have been clarified as mentioned above, it is still elusive if brain functions are influenced by microglial cells having the RAN products.

Aggregated misfolded proteins such as HTT have the general ability to propagate between cells^23^. In line with this phenomenon, we reported that aggregated 13A, 13S and 13L entered PC12 cells and some of them changed their morphology^22,24^. Especially, microglial cells are expected to facilitate such propagation because microglial cells phagocytose cell debris and damaged neurons^25^. Indeed, we have previously shown that fluorescence-labeled polyQ was spontaneously taken up by BV2 microglia cells, which resulted in morphological changes of the cells^17^. Thus, it is likely to predict that the RAN products are also taken up by microglial cells.

In the present investigation, we particularly focused on polyL among the multiple RAN proteins. PolyL encoded by mixed DNA repeats was reported to be more toxic than polyQ in a mammalian cell line^26^, although it is not known if that depends on cell types or that is a general phenomenon. We introduced aggregated polyL peptide into cultured microglia-like BV2 cells and employed morphological analysis. Then, degeneration of neuron-like cells in culture in the presence of the CM from the polyL-incorporating BV2 cells was studied. Finally, brain functions of the mice given the CM were studied.

## Results

### 13A, 13S and 13L induced morphological changes of BV2 microglia with different extent

Among the RAN proteins that are generated from polyQ disease-causative genes, polyC peptide with 13 cysteine repeat was difficult to synthesize^22^, whereas, polyA, polyS, polyL having 13 repeats of the corresponding amino acids (13A, 13S and 13L) could be obtained and aggregated^22,24^. Thus, we sought to study toxicity of microglia having either of the three RAN peptides inside.

**Figure 1:**
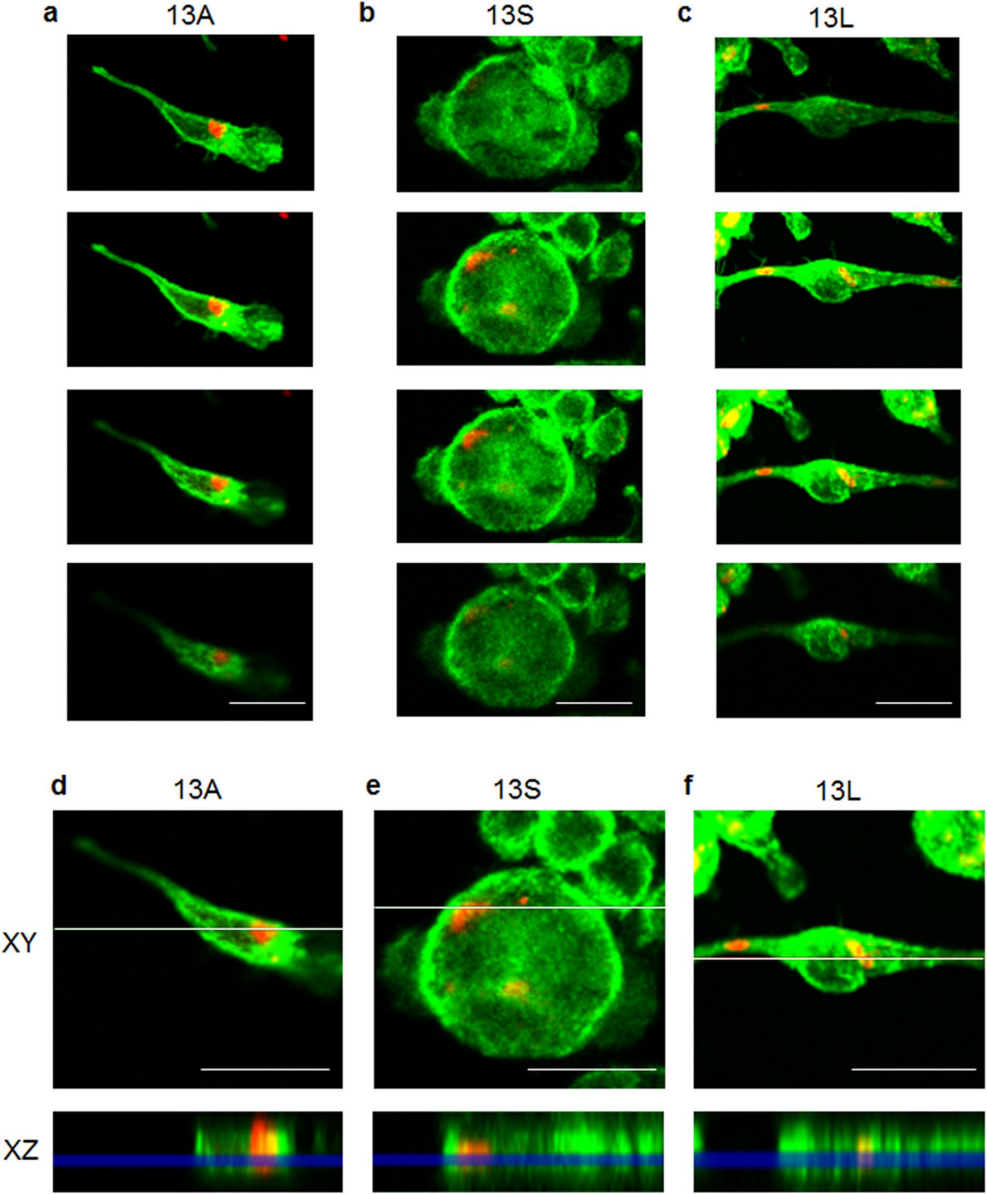
Aggregated 13A, 13S and 13L are incorporated into BV2 microglia cells. Fluorescence z-stack images of BV2 microglial cells with 13A (**a**,**d**), 13S (**b**,**e**) and 13L (**c**,**f**) using confocal laser scanning microscopy. (**a**–**c**) The serial section images of cells having 13A (**a**), 13S (**b**) and 13L (**c**) taken at every 1 µm are shown. (**d**–**f**) Ortho images constructed from the z-stacks. The XZ-axis cross section images (bottom) along the white line in the XY-axis view (top) are shown. 13A (**d**), 13S (**e**) and 13L (**f**) signals (red) are found between cytoplasmic phalloidin signals (green) in the XZ-axis view. Scale bar 20 μm.

We added aggregated 13A, 13S and 13L to culture medium of BV2 microglia at a final concentration of 10 µg/ml. Four days after the addition of the aggregates, serial sectional images by confocal microscopy revealed localization of the 13A (Fig. 1a), 13S (Fig. 1b) and 13L (Fig. 1c) inside the cells (Fig. 1) as seen in PC12 cells^22,24^. Ortho images of the XZ-axis cross section view verified that the 13A (Fig. 1d), 13S (Fig. 1e) and 13L (Fig. 1f) aggregates distributed between cytoplasmic phalloidin signals. These results confirmed that the three aggregates were taken up by microglial cells. Quantification of the cells revealed that 13A, 13S and 13L were taken up by 67%, 65% and 93% of BV2 cells, respectively.

**Figure 2:**
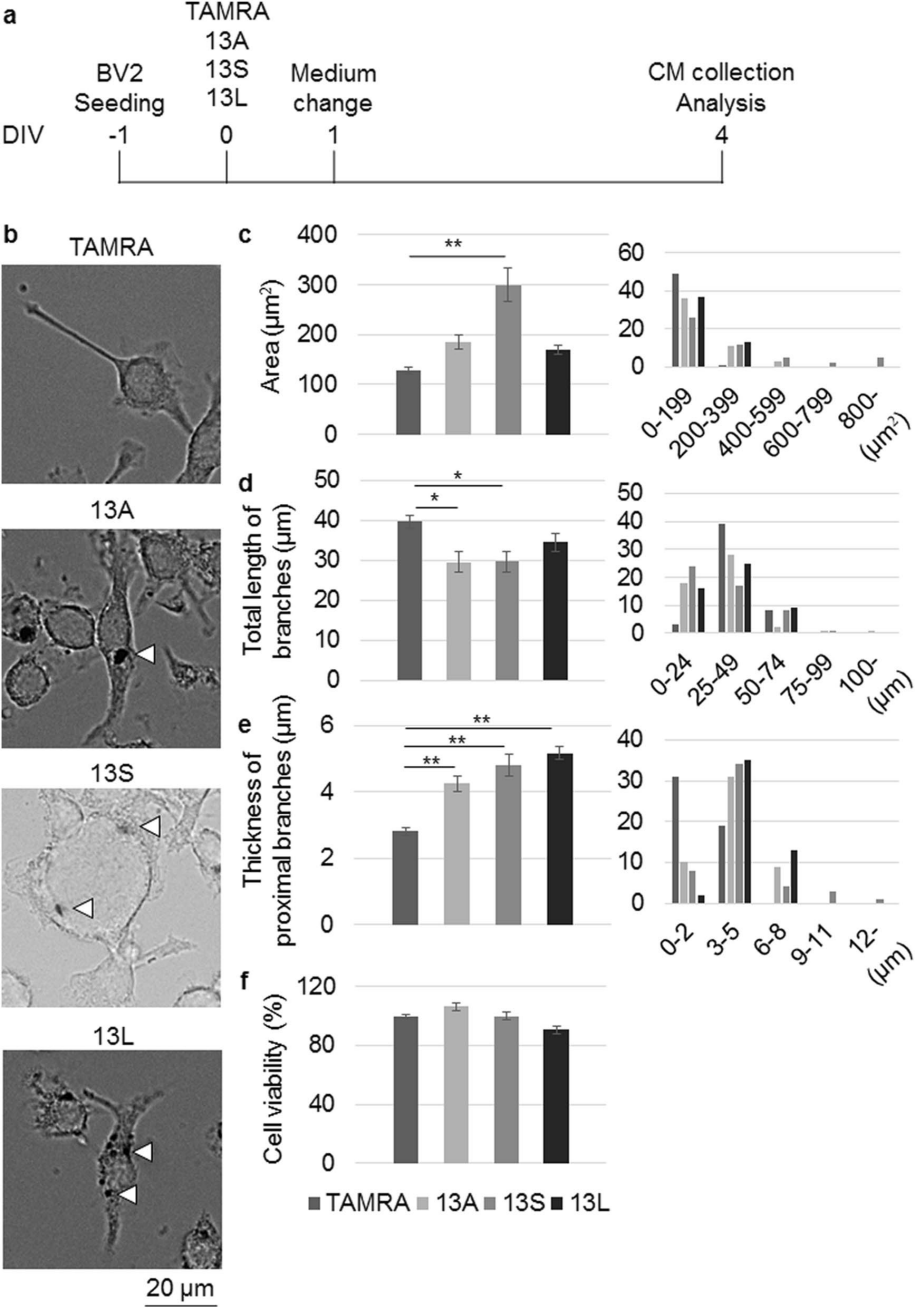
Morphological changes of BV2 microglia by addition of 13A, 13S and 13L. (**a**) Timing of addition of 13A, 13S or 13L and collection of CM. (**b**–**e**) Representative images (**b**) and quantification of the area (**c**), total length of branches (**d**) and thickness of proximal branches (**e**) of BV2 microglia with TAMRA, 13A, 13S or 13L (n = 50 cells, each from 3 independent experiments). Arrowheads indicate aggregates. x-axis and y-axis of histograms (right panels) indicate distribution of the values and numbers, respectively. (**f**) Viability of BV2 cells with TAMRA, 13A, 13S or 13L. The values relative to that of TAMRA are expressed. Error bars represent SE. ANOVA, *p < 0.05; **p < 0.01. Scale bar 20 μm.

We previously reported that microglial cells were changed when the cells phagocytosed polyQ aggregates^17^. To check if 13A, 13S and 13L were also the case, we added the aggregates to medium of BV2 microglial cells. Then, we excluded the aggregates from the medium and cultured the cells 3 days more (Fig. 2a). As the control, we also included TAMRA-treated cells because the three peptides are TAMRA-conjugated. Then, morphology of the cells was quantitatively estimated. For the quantification, we used three parameters: the area of cell body, total length of branches and thickness of proximal branches as previously reported^17^. The area of cell body in 13S- but not 13A- and 13L-treated BV2 microglia was larger than TAMRA-treated cells (Fig. 2b,c). Similarly, total lengths of branches in 13A- and 13S- but not 13L-treated cells were significantly shorter than TAMRA-treated cells (Fig. 2b,d). However, proximal branches were thicker in 13A-, 13S- and 13L-treated cells than TAMRA-treated cells (Fig. 2b,e). Thus, the three aggregates led to changes in morphology but with different shapes each other. In contrast, viability of BV2 cells was not changed in the presence of 13A, 13S or 13L (Fig. 2f).

**Figure 3:**
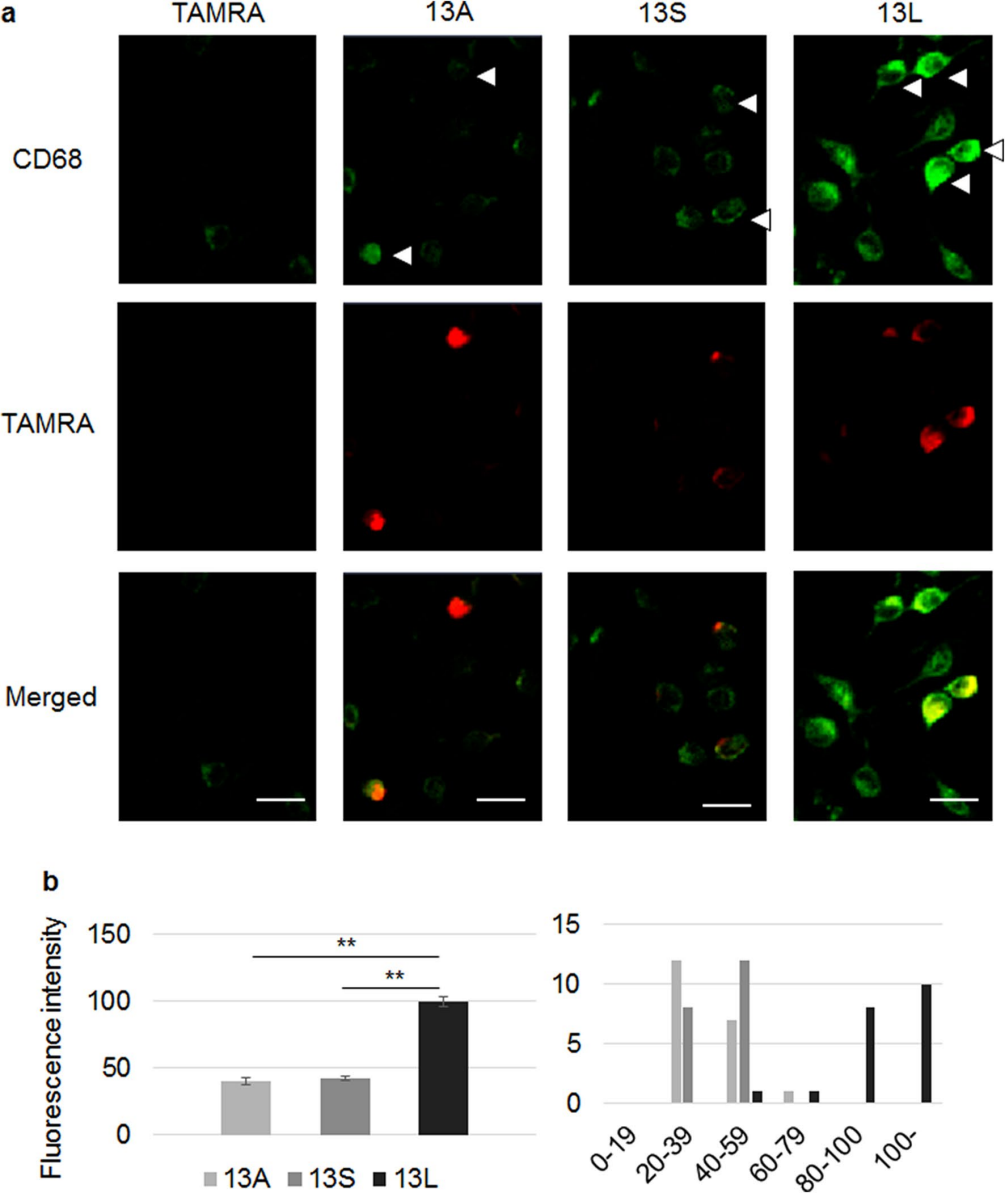
13L induced high CD68 expression in BV2 microglia. (**a**) Immunofluorescence staining of BV2 cells having TAMRA, 13A, 13S or 13L (red) with anti-CD68 antibody (green). Arrowheads indicate BV2 cells having visible large aggregates. (**b**) Intensities of CD68 signals were quantified and were expressed as values relative to that of 13L-treated cells. n = 20 cells each. x-axis and y-axis of a histogram (right panel) indicate distribution of the values and numbers, respectively. Error bars represent SE. ANOVA, **p < 0.01. Scale bars 20 μm.

We then examined state of BV2 cells using an another index. CD68 expression became higher in BV2 cells by addition of LPS^27^, indicating that CD68 expression likely reflects pathological state of microglia. BV2 cells were stained with CD68 antibody and the signal intensities of the cells having visible aggregates were quantified. As shown in Fig. 3a,b, 13L led to significantly higher CD68 expression than 13A and 13S. Faint CD68 signals were also detected in cells without visible large aggregates, probably due to presence of invisible very small aggregates. Thus, 13L likely transformed BV2 cells into pathologically relevant cells.

### CMs derived from 13S- and 13L-treated BV2 microglia induced neurite retraction of PC12 cell

CM includes the factor(s) secreted from cultured microglia-like BV2 cells having the aggregates inside. We previously reported that CM derived from polyQ-treated BV2 microglia induced neurite retraction in differentiated neuron-like PC12 cells^17^. Hence, we decided to culture PC12 cells with CMs collected as shown in Fig. 2a.

**Figure 4:**
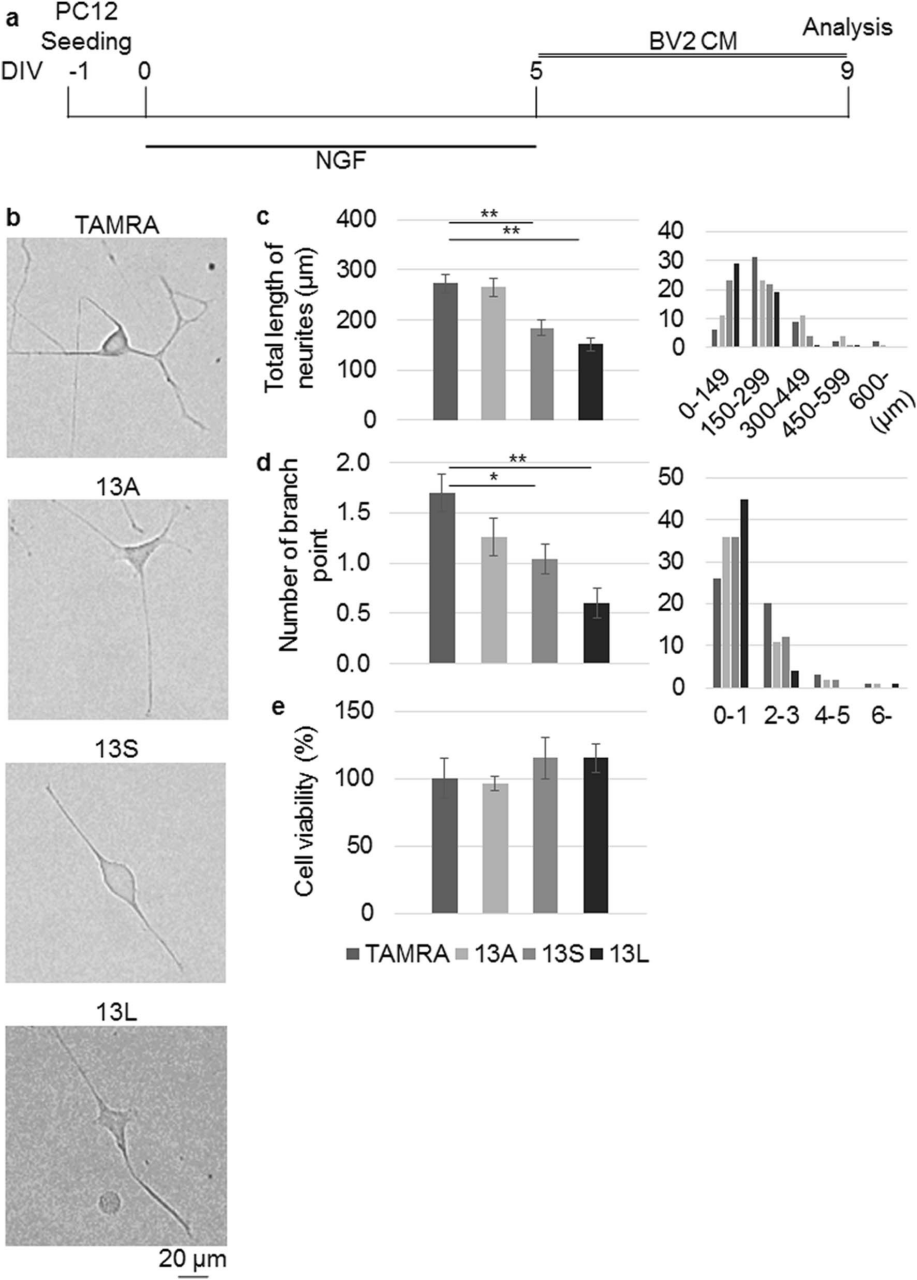
CM derived from BV2 microglia treated with 13S and 13L induce degeneration of differentiated PC12 cells. (**a**) Timing of addition of CM from BV2 microglia having TAMRA, 13A, 13S or 13L. (**b**–**d**) Representative images (**b**) and quantification of the total length of neurites (**c**) and the number of branch point (**d**) of differentiated PC12 cells cultured in the CM (n = 50 cells, each from 3 independent experiments). x-axis and y-axis of histograms (right panels) indicate distribution of the values and numbers, respectively. (**e**) Viability of PC12 cells in the presence of CMs from BV2 cells with TAMRA, 13A, 13S or 13L. The values relative to that of TAMRA are expressed. Error bars represent SE. ANOVA, *p < 0.05; **p < 0.01. Scale bar 20 μm.

To study degeneration of neuron-like PC12 cells in the presence of CMs, neurites of PC12 cells were needed to be fully elongated before the addition of CMs. To this end, the cells were initially cultured for 5 days in the presence of NGF. NGF was removed on day 5 and the cells were further cultured for 4 days in the presence of CMs (Fig. 4a). To see degeneration of cells, retraction of neurites was estimated using 2 indices; total length of neurites of cells and number of branch point of neurites. 13S-CM and 13L-CM induced significantly shorter neurites than that by TAMRA-CM (Fig. 4b,c). However, 13A-CM did not lead to shorter neurites (Fig. 4b,c). Numbers of branch point of the neurites were significantly fewer in 13S-CM- and 13L-CM-treated PC12 cells than in TAMRA-CM-treated cells. However, 13A-CM did not alter the number (Fig. 4b,d). Again, viability of PC12 cells was not changed in the presence of 13A-CM, 13S-CM or 13L-CM (Fig. 4e).

### 13L-treated BV2-CM injected in PnC reduced startle amplitude in mice

Given the degeneration of neuron-like PC12 cells by CMs derived from 13S-treated and 13L-treated BV2 microglial cells, we sought to address the question whether the CMs also affect brain functions in mice. Since the ventricular system widely spreads in the brain, the CMs were injected into right lateral ventricle and did a battery of behavioral tests the next day.

**Figure 5:**
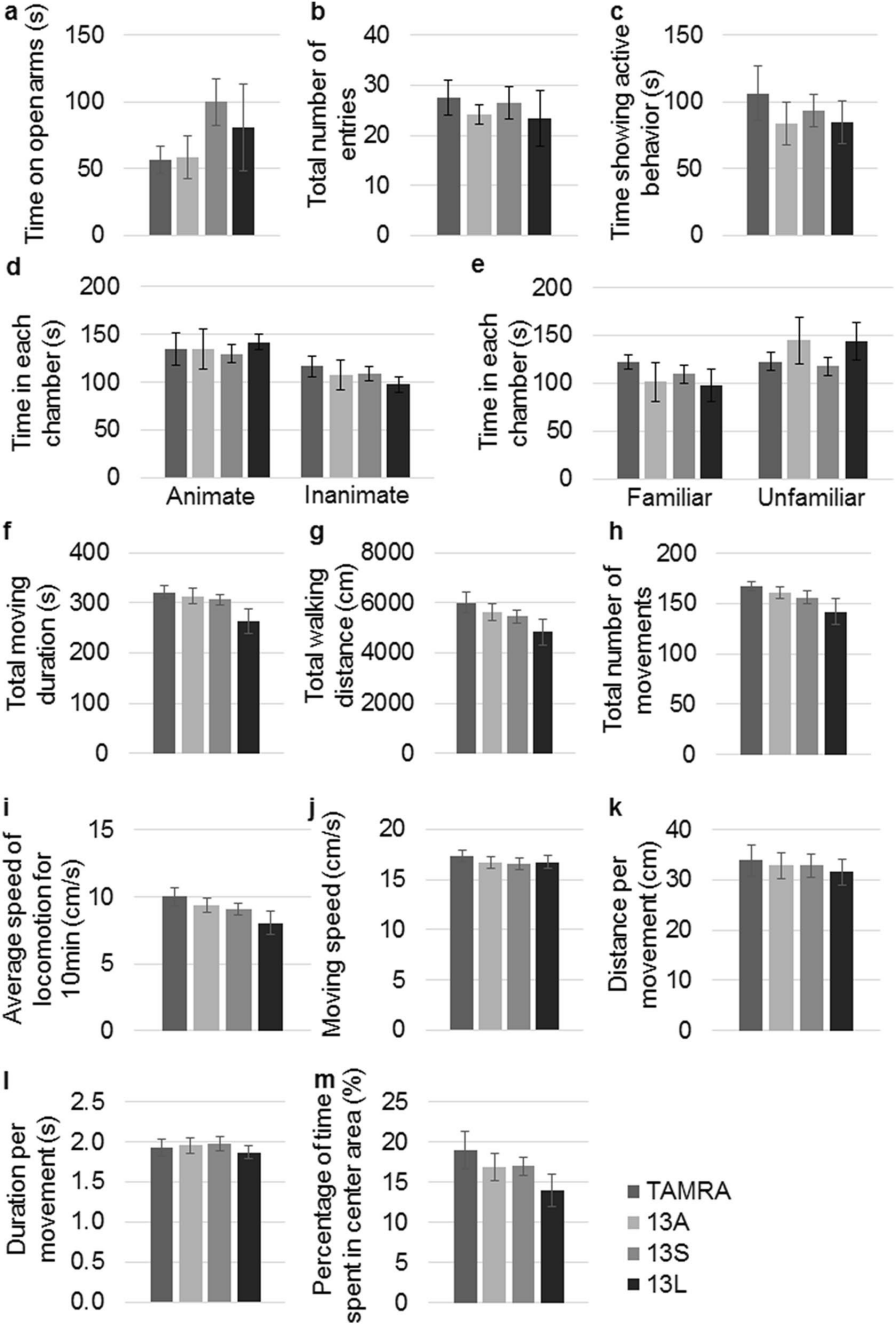
Injection of CM derived from BV2 microglia treated with 13A, 13S or 13L into lateral ventricle does not alter anxiety, active behavior, social behavior and spontaneous motor activity. (**a**,**b**) Elevated plus maze test. Time on open arms (**a**) and total number of entries into the arms (**b**) were measured in mice injected with CM from TAMRA-, 13A-, 13S- or 13L-treated BV2 cells. (**c**) Resident-intruder test. Total time showing active behavior (approaching, chasing, sniffing) against intruder mouse was measured in mice. (**d**,**e**) Three chamber social test. The times spent in the chamber with (animate) and without (inanimate) a mouse (**d**) and the times spent in the chamber with the same mouse (familiar) and a novel mouse (unfamiliar) (**e**) were measured. (**f**–**m**) Open field test. Total moving duration (**f**), total walking distance (**g**), total number of movements (**h**), average speed of locomotion for 10 min (**i**), moving speed (**j**), distance per movement (**k**), duration per movement (**l**) and percentage of time spent in the center area (**m**) were measured. n = 10 each, 5 males and 5 females. Error bars represent SE.

We recently showed that injection of 13S and 13L themselves into the lateral ventricle altered anxiety and depression or stress-coping behavior in mice, as proved by elevated plus maze and forced swim tests, respectively^22^. Therefore, we carried out an elevated plus maze test using 13A-, 13S- and 13L-CMs. The mouse was first put on the center area and was allowed to freely enter the open and closed arms. The mice with 13A-, 13S- and 13L-CM in the ventricle spent time comparable to that in mice given TAMRA-CM (Fig. 5a). Similarly, spontaneous motor activities on the maze were not different among the groups because no differences in total number of entries into the arms were found (Fig. 5b). These results indicate that any CMs did not affect anxiety. As a behavioral test for a different kind of emotion, we tested active behavior using resident-intruder test. The male mouse that has been a resident in a cage shows active behavior (approaching, chasing or sniffing) against a male intruder mouse. The total time for the resident mice showing active behavior were not different among the 4 groups (Fig. 5c). The next test we employed was three chamber test. The time a subject mouse spent in the chamber with a mouse was not different among the groups (Fig. 5d), suggesting no difference in the social behavior. Then, a novel mouse was added to the cage that had been empty in the last trial, leaving the old mouse in another chamber. Likewise, the time in the chamber with the novel mouse was not different (Fig. 5e), indicating no difference in social memory.

Injection of 13S and 13L themselves into the lateral ventricle did not essentially change open-field performances^22^. Similarly, when we checked general motor activity in an open-field after injection of the CMs into the lateral ventricle, there were no differences in total moving duration (Fig. 5f), total walking distance (Fig. 5g), total number of movements (Fig. 5h), average speed of locomotion for 10 min (Fig. 5i), moving speed (Fig. 5j), distance per movement (Fig. 5k), duration per movement (Fig. 5l) and percentage of time spent in center area (Fig. 5m) among TAMRA-, 13A-, 13S- and 13L-CM injected mice.

**Figure 6:**
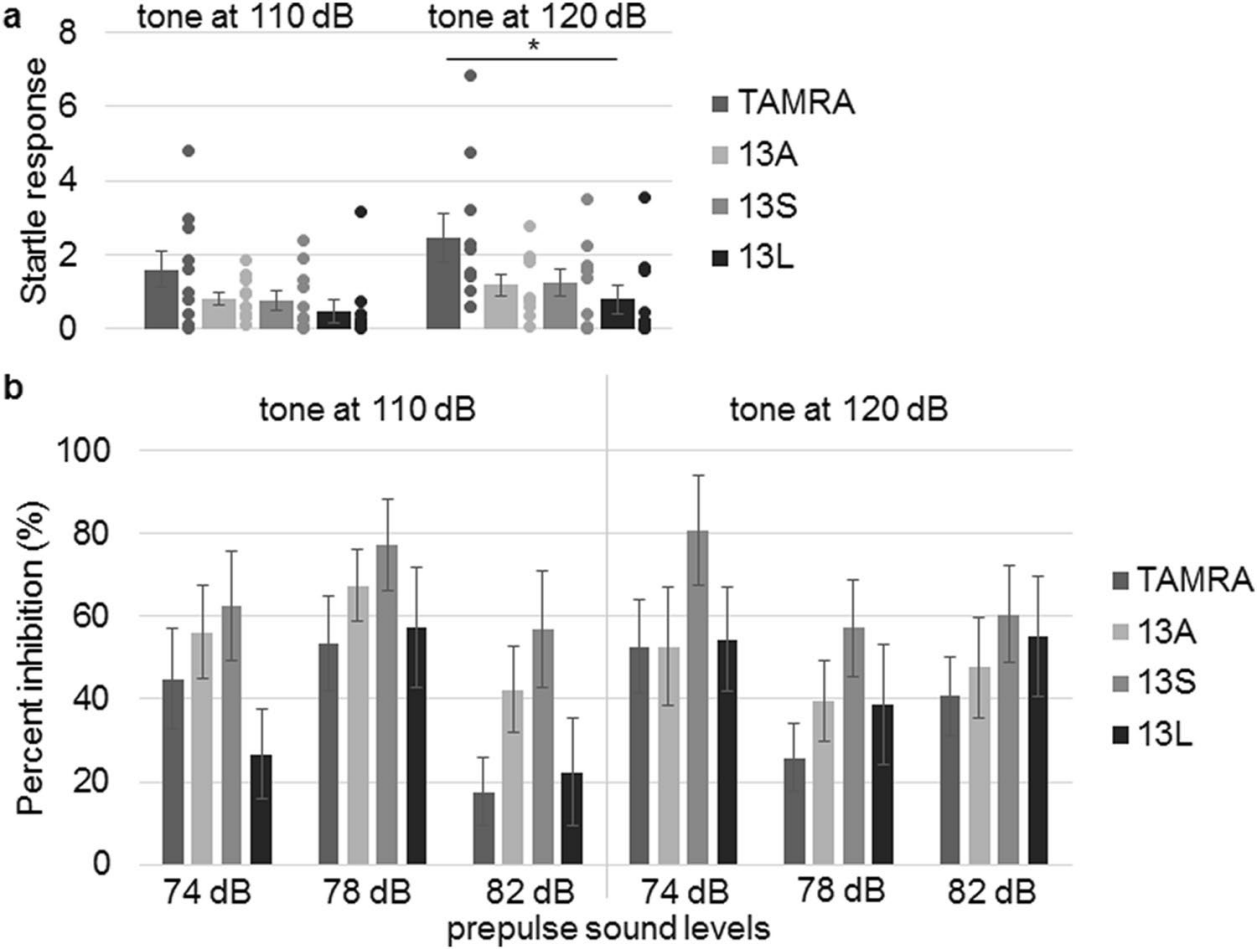
Injection of CM derived from 13L-treated BV2 microglia into lateral ventricle reduces startle response at 120 dB. Acoustic startle responses at 110 or 120 dB (**a**) and inhibition of the responses by prepulses (74, 78 or 82 dB) (**b**) in mice to which CM from BV2 microglia having TAMRA, 13A, 13S or 13L (n = 10 each, 5 males and 5 females) was injected. Error bars represent SE. ANOVA, *p < 0.05.

Startle response is a fast twitch of muscles elicited by sudden and intense stimuli. The physiological significance of this response is hypothesized to protects animals from injury by a predator^28^. Acoustic, tactile and visual stimuli can evoke the response. We tested acoustic startle response after sound pressure level at 110 and 120 dB. There were no significant differences in startle response at 110 dB. However, 13L-CM, but not 13A- and 13S-CMs, evoked significantly smaller startle response than TAMRA-CM at 120 dB (Fig. 6a).

The magnitude of acoustic startle response is diminished by preceding non-startling acoustic stimulation (prepulse)^29^. This is called prepulse inhibition (PPI), which theoretically reflects sensorimotor gating. The prepulse and startling sound levels used in the experiment were 74, 78, 82 dB and 110, 120 dB, respectively. No significant differences in PPI were detected by any combination of prepulses and startling sounds among the 4 groups (Fig. 6b). Collectively, only 13L-BV2-CM led to reduced startle response at 120 dB.

**Figure 7:**
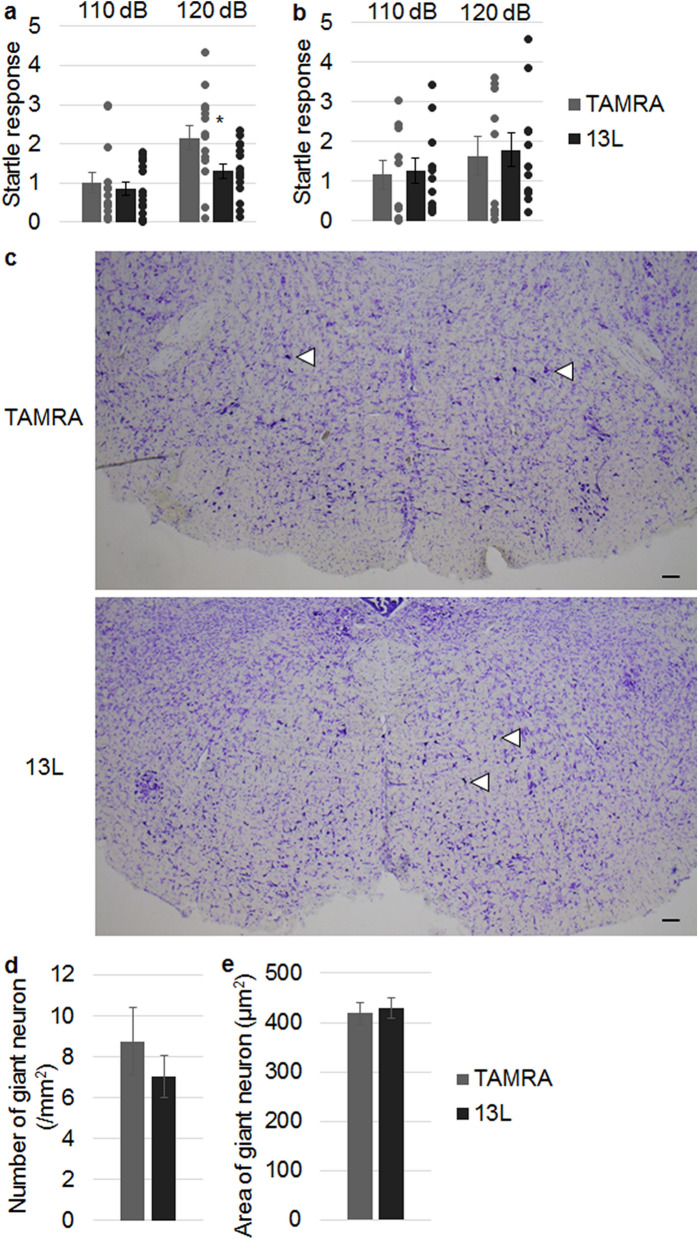
Reduced startle response in mice to which CM derived from 13L-treated BV2 microglia was injected into PnC. (**a**) Startle responses in mice to which CM from TAMRA-treated (n = 14, 9 males and 5 females) or 13L-treated (n = 15, 10 males and 5 females) BV2 microglia was injected into the PnC. (**b**) Startle responses were also measured in mice to which heat-denatured BV2-CMs were injected into the PnC. n = 10 (5 males and 5 females) and n = 11 (5 males and 6 females) for TAMRA-treated BV2-CM and 13L-treated BV2-CM, respectively. (**c**–**e**) The number (**d**) and size (**e**) of giant neurons (arrowheads in panel **c**) in the PnC of Nissl-stained coronal brain sections from TAMRA-CM- and 13L-CM-injected mice were compared (data were obtained from 6 male mice, each). Scale bar 100 µm. Error bars represent SE. ANOVA, *p < 0.05.

Since 13L-CM in the lateral ventricle altered only startle response, we searched for the responsible brain region. The neuronal pathway controlling acoustic startle response includes the auditory nerve, the ventral cochlear nucleus, the dorsal nucleus of the lateral lemniscus, the caudal pontine reticular nucleus (PnC) and spinal motor neurons^30^. Among them, neurons located in the PnC play key roles in primary acoustic startle pathway^28^. We measured startle response 1 day after injection of 13L-BV2-CM into the PnC. As shown in Fig. 7a, magnitude of startle response at 120 dB was lower in 13L-CM-injected mice than TAMRA-CM-injected mice. In contrast, a sound level at 110 dB did not elicit the difference. However, the startle response at 120 dB was not different between the two groups when the CMs heated at 100 °C for 10 min were injected into the PnC (Fig. 7b). Therefore, the responsible factor(s) released from BV2 microglial cells might be proteins because the heat denaturation of proteins is essentially irreversible at temperatures higher than 80 °C^31,32^. The results after injection of the CM into the PnC (Fig. 7a) were same as those after injection of the CM into the lateral ventricle (Fig. 6a). Using mice given 13L-CM in the PnC, we counted the number of the giant neurons in the PnC because the neurons are involved in neural circuit of acoustic startle response^33,34^. The number was not different between TAMRA-CM-injected mice and 13L-CM-injected mice (Fig. 7c,d). Likewise, the size of the neurons was identical between the 2 groups (Fig. 7c,e). These results are in line with the finding that 13L-CM did not change viability of PC12 cells (Fig. 4e).

## Discussion

Theoretically, endogenous RAN proteins could be translated by polyQ-deaseses-causative genes in the microglial cells in the brain, and also the aggregated RAN products in damaged neurons could be phagocytosed by microglia. Therefore, we mimicked the situation where aggregated RAN proteins are located in the microglial cells by introducing pre-aggregated polyA, polyS and polyL peptides in microglia. The three peptides have common flanking sequences (KKW and KK) at both sides of the core repeats (A_13_, S_13_ and L_13_). Since the three peptides have a fluorophore TAMRA, we also treated microglial cells with TAMRA as the control. Scrambled peptides with different order of amino acids are generally used to determine the specific effects of the sequences of peptides. However, we could not prepare the single scrambled peptide because we used three different peptides. Nevertheless, we could suggest specific contribution of the sequences of polyL and polyS to functions of the microglia because CM from polyA-treated microglia did not show any morphological changes of PC12 cells. Therefore, polyA works as the negative control.

Prion-like propagation of proteins is an intriguing phenomenon in which aggregated proteins spontaneously transmit from a cell to other cells. We found uptake of aggregated polyA, polyS and polyL peptides by BV2 microglial cells. Misfolded proteins that are responsible for neurodegenerative disorders including aggregated HTT with expanded polyQ^19^ have such ability^20^. As the mechanisms of the transfer of polyQ-containing proteins, an interaction of polyQ aggregates with the cell membrane^23^ and clathrin-dependent endocytosis^31^ have been suggested. We have recently shown that aggregated polyA, polyS and polyL peptides were also taken up by neuron-like PC12 cells^22,24^. The entry of the polyS and polyL into PC12 cells seems to use endocytosis because of the presence of the invaginated pits from the cell membrane containing the aggregates inside them, as proved by electron microscopic analysis^22^. In neurodegenerative disorders, the microglial cells are changed and migrate to the lesion sites where the cells phagocytose damaged neurons and cell debris^25^. Phagocytosis is a type of endocytosis but uses clathrin-independent mechanism^35^. There are multiple types of receptors to initiate microglial phagocytosis such as Toll-like receptors with a high affinity to microbial pathogens and triggering receptor expressed on myeloid cells 2 (TREM-2)^25^. Regarding phagocytosis of polyQ-containing protein, prion-like transmission of neuronal HTT aggregates to phagocytic glia was observed in the Drosophila brain^36^. Uptake of aggregated polyA, polyS and polyL by BV2 microglia in this study raises a possibility that not only polyQ proteins themselves but their RAN products have a potential to exert the prion-like transmission to phagocytic microglia. There is a need to specify the mechanisms that enable phagocytosis of polyA-, polyS- and polyL-containing proteins. It is of particular important to determine whether phagocytosis of polyA, polyS and polyL by microglia uses a common receptor.

The nature of microglia is closely related to its morphology. Pathogenic insults change the appearance of microglia from a highly ramified morphology to an amoeboid shape^37–39^. We therefore examined morphological changes of BV2 microglia after uptake of 13A, 13S and 13L. We applied three indices that reflect the shape of BV2 microglia. When microglial cells display amoeboid shape, large area of the cell, short length of branches and thick proximal branches are likely observed. The morphological changes by 13A, 13S and 13L were not identical. 13S led to the most prominent change in the morphology, showing larger area of cell body, shorter length of branches and thicker proximal branches. In contrast, 13A brought only shorter length of branches and thicker proximal branches with no difference in the area of the cell. Likewise, 13L induced only thicker proximal branches. Thus, biggest morphological changes were seen in 13S-incorporating BV2 microglia rather than 13A- and 13L-incorporating cells. However, CD68 expression level was highest in BV2 cells having 13L. Thus, the morphological changes and CD68 level seem not to reflect a same state of microglia.

Consistently, toxicity of CMs from 13S- and 13L-incorporating microglia to PC12 cells was almost identical. 13S-CM and 13L-CM induced both shorter length of neurite and fewer number of branch point in PC12 cells, whereas, 13A-CM did not induce any morphological changes. Thus, degree of morphological changes of BV2 microglial cells seems not to be completely correlated with the toxicity of factor(s) released from BV2 microglial cells. Both short length of neurites and few number of branch point were observed in PC12 cells treated with CM from polyQ-incorporating microglia^17^. Thus, 13L-incorporating microglia and those having polyQ might release same factor(s) that are toxic to neurons. It was reported that CM from microglia from human HD did not overtly affect the viability of striatal neurons from human pluripotent stem cell^40^. In line with this observation, viability of PC12 cells was not changed in the presence of 13L-BV2-CM. However, we cannot exclude a possibility that enhanced new cell genesis by the CM hid the higher cell death in the in vitro viability assay.

TNFα and IL-6 are candidates of the toxic factor(s) released from 13L-treated BV2 microglial cells based on the following observations associated with polyQ diseases. ATXN1[82Q] mice, a model for SCA type 1, showed significantly higher TNFα, MCP-1 and IL-6 mRNA levels in the cerebellum at 12 weeks old^41^. Even at 4 weeks old, the mice showed significantly higher TNFα and MCP-1 mRNA levels, but that of IL-6 was not significantly higher^41^. In humans, ELISA assay clarified elevated levels of TNFα and IL-6 in the plasma from patients with moderate HD^42^. Moreover, IL-6 mRNA level was elevated in the striatum from HD individuals^3^. As autonomous microglial changes by mutant HTT, increased TNFα and IL-6 mRNA levels were observed^3^. Similarly, TNF and IL-6 were elevated in HD pluripotent stem cell-derived microglia with longer polyQ repeat after stimulation with LPS and IFN^40^. However, the amount of TNFα in 13L-BV2-CM (117 ± 42 pg/ml) was not different from that in TAMRA-BV2-CM (107 ± 13 pg/ml) (p = 0.08) and IL-6 levels were below the detection limit. Thus, the toxic factor(s) that are released from 13L-treated BV2 cells have not been currently specified. The undefined factor(s) might finally induce reactive oxygen species. HD pluripotent stem cell-derived microglia released high levels of reactive oxygen species, which can directly cause damage to neurons by oxidative effects on the lipids, nucleic acids and proteins^40^. Indeed, addition of a low concentration of hydrogen peroxide induced neurite degeneration without cell death in culture^43^. Whatever the case, the finding that 13L-CM, but not 13A-CM and 13S-CM, in the ventricle specifically lowered startle response clearly indicates that the degree of toxicity of the released factors is differential among the three RAN products. Thus, inhibition of the release of polyL-specific factors by microglia likely prevents the effect on startle response. In BV2 microglial cells, part of phagocytosed 13L co-localized with a lysosome marker LAMP1 (Supplementary Fig. S1 online), indicating that 13L might potentially undergo lysosomal degradation. Nevertheless, abundant 13L aggregates were located in the cytoplasm of BV2 cells. Thus, 13L that did not undergo lysosomal degradation in endogenous microglia in the mouse brain likely changes the functions and promotes the release of the polyL-specific factors from microglia. The 13L-CM taken at early time point (from day 2 to day 4 after the addition of 13L) changed the functions in vitro and in vivo (Fig. 2). The 13L-CM collected at later time point (from day 5 to day 6) also induced same morphological changes in PC12 cells (Supplementary Fig. S2 online). Therefore, the factor(s) seem to be released at least until day 6 in BV2 cells.

We recently reported that injection of 13L itself into the lateral ventricle changed brain functions in mice^22^. The behavioral tests were carried out the next day after 13L injection. To compare the results^22^ with those after injection of 13L-BV2-CM, we also did behavioral tests the following day after injection of the CM in this study. One day after injection might not be enough to obtain full recovery from the operation. Therefore, we cannot exclude a possibility that behavioral results might be different at later time points after the injection.

The application of 13L itself and that of 13L-BV2-CM led to different results in vitro and in vivo. An increased anxiety as evidenced by elevated plus maze test was recognized in mice to which 13L itself was injected into the lateral ventricle^22^. By contrast, when 13L-BV2-CM was injected into the lateral ventricle of mice, their performances on the maze did not differ from TAMRA-BV2-CM-injected mice. Therefore, the increase in anxiety seen in 13L-injected mice could be ascribable to toxicity of aggregates to neurons in a microglia-independent manner. In contrast, addition of 13L itself to neuron-like PC12 cells did not induce neurite retraction^22^, whereas, 13L-BV2-CM did cause the retraction in vitro. It is possible that resident microglial cells in the brain having endogenous polyL-containing RAN proteins impair functions of adjacent neurons in a non cell-autonomous manner. In line with this assumption, resident microglia in the PnC captured 13L in the mouse brain. Thus, it is plausible to predict that polyL and polyL-containing microglia differentially affect neuronal functions in the brain.

Regarding startle response of model mice for polyQ diseases, the R6/2 transgenic mouse line, a model for HD, exhibited comparable startle levels after acoustic stimuli at 105 dB and 120 dB to wild-type mice during young stages. However, the levels became lower in the model mice at 12.5 weeks of age^44^. Likewise, the startle magnitude was higher at some ages but was lower at the different ages in the HdhQ92 line, a knock-in model mouse for HD^45^. We showed an involvement of CM from 13L-treated BV2 microglia in startle response. Our data implies that not only polyQ in neurons but its RAN product in microglia decreases acoustic startle response. However, the lower response was seen at 120 dB but not at 110 dB. Therefore, the functional defects by 13L-CM that are closely linked to startle response might be subtle, thereby making a difference specifically after a strong startling stimulus. Iba 1-positive microglia captured injected 13L in the PnC (Supplementary Fig. S3), indicating that endogenous microglia can recognize polyL. However, it has not been determined if the capture in vivo leads to lower startle response as seen by injection of 13L-BV2-CM.

Subpopulation of giant reticulospinal neurons in the PnC receive direct acoustic input from the central auditory pathway. Then, the PnC neurons project to spinal motor neurons and are therefore regarded as sensorimotor interfaces for the components of acoustic startle response^28^. The lower startle response observed when 13L-CM was injected into the PnC implies that either axon terminals of neurons of the central auditory system in the PnC or giant reticulospinal neurons themselves in the PnC was impaired. Although we could not find differences in the density and size of the giant neurons, the subtle changes might be present given the changes of neurites in PC12 cells. Inspection with electron microscope will clarify this point in future. Alternatively, neuronal activity of the giant neurons after the startle stimuli might be changed.

Although R6/2 and YAC128 mice, models for HD, developed deficits in PPI of startle reflex^44,46^, CM from 13L-treated BV2 microglia in the lateral ventricle did not lead to defective PPI. The brain regions responsible for modulation of startle response by prepulse are broadly distributed. The regions include multiple regions such as pedunculopontine tegmental nucleus, laterodorsal tegmental nucleus, locus coeruleus and substantia nigra, pars reticulate^47^. Thus, the PnC neurons might be functionally vulnerable to 13L-BV2-CM than other regions. The sensitivity of each brain region other than the PnC to 13L-BV2-CM will be determined by behavioral tests using mice given the CM into each region in near future.

## Methods

### Peptides

The sequences of the synthesized TAMRA-labeled 13A, 13S and 13L were 5-TAMRA-KKWAAAAAAAAAAAAAKK-NH2, 5-TAMRA-KKWSSSSSSSSSSSSSKK-NH2 and 5-TAMRA-KKWLLLLLLLLLLLLLKK-NH2 (GL Biochem, Shanghai, China), respectively. The purity of 13A and 13S were more than 95%. However, that of 13L was 75% because purification of the peptide was difficult. The stock solutions of the peptides were prepared by dissolving the powder in a 1:1 mixture of trifluoroacetic acid and hexafluoroisopropanol. The appropriate conditions to induce aggregation of the peptides were determined in our previous literature^22,24^. 13L had highest aggregation property among the three peptides. It aggregated immediately after addition into culture media at a concentration of 10 µg/ml. By contrast, 13A and 13S needed long time incubation in aqueous solution for aggregation. Prior to addition to culture media, 13A and 13S were incubated in aqueous solution (1 mg/ml) for 7 days at 37 °C without shaking and for 2 days at 37 °C with shaking at 206 rpm/min., respectively, to induce aggregation.

### Preparation of CM

BV2 microglial cell line was kindly provided by Dr. Choi (Korea University). The cell line was cultured in DMEM containing 10% fetal bovine serum (FBS) and 1% penicillin–streptomycin mixed solution at 37 °C with 5% CO2. BV2 cells were plated on micro cover glass in 6-well plates at density of 8.0 × 10^4^ cells per well. 13A, 13S and 13L were applied to the culture medium to be a final concentration of 10 µg/ml. The next day, the medium was removed and cultured cells were washed with PBS once to remove the peptides. Then, the cells were further cultured for 3 days more in the same culture medium without peptide to collect CM. As a different protocol, the culture medium was changed 4 days after the addition of 13L to BV2 cells. Then, BV2 cells were cultured for 2 days more to collect CM (Supplementary Fig. S2a). ELISA assay was performed using ELISA MAX Deluxe Set Mouse TNFα and IL-6 (BioLegend, San Diego, CA) according to the manufacturer’s instruction.

### PC12 cell culture in the presence of CM

PC12 cell was purchased from RIKEN BRC. PC12 cells were plated on micro cover glass coated with laminin in 24-well plates at density of 5 × 10^3^ cells per well. Differentiation of the cells was induced for 5 days in the presence of 50 ng/ml NGF in DMEM containing 1% FBS and 0.25% BSA. Then, the cells were further cultured for 4 days in the presence of CM without NGF.

### Quantification of cultured cells and mouse neurons

Morphology of cultured cells and mouse neurons were quantified using Image J software as previously reported^22^. The original images were acquired by the software and the actual scale in the images was reflected to the acquired images. Then, length and area of interest were measured by tracing them using tools of the software. Because the length less than 1 µm was difficult to trace, the processes longer than 1 µm were measured. The area of cell body, total length of branches and thickness of proximal branches of BV2 microglia, the total length of neurites and number of branch points of the neurites of PC12 cells and area of cell body of the PnC neurons in mice were measured. Intensity of CD68 signals in BV2 cells was also measured using Image J software. Viability of cultured cells was determined using Cell Counting Kit-8 (Dojindo Molecular Technologies Inc., Rockville, MD) according to the manufacturer’s instruction.

### Mice

All experimental protocols were approved by the Animal Resource Committees of Gunma University. All methods were carried out in accordance with relevant guidelines and regulations (NIH) and were reported in accordance with ARRIVE guidelines for the reporting of animal experiments. The number of mice used for experiments was bare minimum to obtain reliable data and we made every effort to minimize the suffering of mice during experiments. Mice were kept in specific pathogen-free conditions in a room where the temperature and light/dark cycle were constant (23 °C and 12 h, respectively). Male and female ICR mice 2–3 months of age were anesthetized with isoflurane and were fixed by stereotaxic instrument. Then, the mice received injection of CM. Two µl of CM or 13L (100 µg/ml) was injected into the PnC (AP − 5.35 mm; ML + 0.5 mm; DV − 5.6 mm)^48^. Five µl of CM was injected into the right lateral ventricle (AP + 0.2 mm; ML + 0.8 mm; DV − 2.5 mm)^22^. Behavioral tests were performed on the following day.

### Imaging

Fluorescent staining was done essentially as described previously^17,22,49^. BV2 cells were fixed with 4% paraformaldehyde (PFA). Mice were also transcardially infused with 4% PFA. After postfixation with same fixative solution overnight and dehydration with 30% sucrose in PBS, coronal brain sections 25 µm in thickness were prepared using cryostat.

The cytoplasm of BV2 cells was stained with Phalloidin-iFluor 488 or 647 conjugate (Cayman Chemical, Ann Arbor, MI) that binds to actin.

For immunofluorescence staining for anti-CD68 antibody (Abcam, Cambridge, UK) and anti-LAMP1 (Merck, Boston, MA) antibody, BV2 cells were incubated with the primary antibodies for 1 h in RT and overnight at 4 °C, respectively. Then, the cells were incubated with Alexa fluor 488-labeled secondary antibody for 1 h in RT. When anti-Iba1 antibody (Gene Tex, Irvine, CA) was used as primary antibody, 2 N HCl was applied to the brain sections for 10 min prior to the addition of the primary antibody overnight at 4 °C. Then, the sections were incubated in HRP-labeled secondary antibody solution for 1 h in RT. Finally, tyramide 488 solution (Thermo Fisher Scientific, Waltham, MA) was applied for 10 min in RT. The fluorescent signals were detected using LSM 880 confocal microscope (Zeiss, Oberkochen, Germany). When the confocal images were acquired, the frame size was 512 × 512 pixels and the scan time was 7.45 s. Line mode and bit depth of 8 bit were applied for averaging. The serial z-stack images of BV2 microglial cells at every 1 µm were also obtained by the confocal laser scanning microscopy.

Nissl staining of brain sections was also carried out essentially as reported previously^22^. The sections were stained with 0.1% cresyl violet solution for 20 min at 60 °C. The visible images were taken by BZ-9000 microscope (Keyence, Osaka, Japan) or ECLIPS 80i microscope (Nikon, Tokyo, Japan).

### Behavioral tests

Resident-intruder test was performed essentially as described previously^52^. An intruder mouse that had not been a resident in the cage was put in the cage where a target mouse with the same sex has been a resident for 2 days. The total time the resident mice was showing active behavior (approaching, chasing, sniffing) against the intruder mouse during 5 min was measured.

Size of the box used for three-chambered social approach task (O’HARA & CO., LTD., Tokyo, Japan) is 20 × 40 × 23 cm. There are three chambers (left, center, right) with an equal size in the box. Mice can enter each chamber through the entrances (5 × 3 cm) between the chambers. Each subject mouse was first put in the center chamber and was allowed to move for 5 min (first trial). After 5 min, the subject mouse was taken out. After a different mouse was placed in the cage in one side of the chamber, the subject mouse was again put in the center chamber and the mouse behavior was automatically measured for 5 min (second trial). Social approach was estimated by the time subject mouse spent in the chamber with the mouse. Then, third trial was conducted by adding a novel mouse in the cage that had been empty in the second trial for 5 min, leaving the old mouse in another chamber. Social memory was evaluated by the time subject mouse spent in the chamber with the novel mouse.

The elevated plus maze test was performed as previously reported^22^. Two open and two closed arms of the maze were 25 cm long and 5.5 cm wide. The closed arms but not open arms had transparent walls with 14.5 cm in height at both sides. The center area of the maze was 5.5 cm × 5.5 cm. The maze was elevated above the ground (55 cm). Mice were first placed on the center area facing an open arm. Entering into the arms was defined when all four paws were on the arms. The total time spent in the open arms and total number of entries into the 4 arms were measured during 10 min.

The open field test was carried out as previously reported^24^. The apparatus (50 cm × 50 cm × 50 cm, O’HARA & CO., LTD., Tokyo, Japan) automatically measured spontaneous locomotion of mice for 10 min. The floor of the open field was covered with black paper to detect movement of the white mouse. Mice were initially placed in the center area and were allowed to walk freely. The parameters measured were duration of movement during test session, total walking distance, total number of movements, average speed of locomotion during test session, speed during movement, walking distance per movement, duration per movement and the percentage of time the mice stayed in the center area.

The acoustic startle response and PPI of the response were recorded using an acoustic startle reflex measurement system (O’HARA & CO., LTD., Tokyo, Japan). Mice were put into a cylinder which was placed on an acceleration sensor in a chamber. A loudspeaker in the chamber produced sounds as the startle stimulus (40 ms) and the software automatically detected the startle responses. For mice given CM into the lateral ventricle, prepulses (20 ms) with 74, 78 or 82 dB was applied 100 ms before the startling acoustic stimuli. Startle responses at 110 and 120 dB and six combinations of PPI (74–110, 78–110, 82–110, 74–120, 78–120, 82–120 dB) were presented 6 times in a pseudorandom order. The intensities of startling stimuli applied (110 and 120 dB) were chosen because the tone at 120 dB was extensively used for 11 inbred strains of mice^50^ and the startle response of ICR mice became big using tones at 110 dB and 120 dB compared to those at 80–100 dB^51^. The prepulse levels (74, 78, 82 dB) were chosen because 74–90 dB were applied for the 11 inbred strains^50^ and 73–86 dB were used for ICR mice^51^. The averaged value after each stimulus type was calculated for each mouse. The interval time of each trial ranged from 10 to 20 s.

The behavioral tests using mice given CM in the lateral ventricle were done in the following order with intervals of 10 min; resident-intruder test, three chamber test, elevated plus maze test, open field test, startle response and PPI. Different sets of mice were used for startle responses of mice given 13L-CM in the PnC. All behavioral tests after injection into the lateral ventricle were done using 5 males and 5 females, each group. For startle responses after injection into the PnC, 5–10 males and 5 or 6 females per groups were used.

### Statistical analysis

The values expressed are the mean in the graphs. The error bars represent SE. Statistical significances were examined using one-way ANOVA except three chamber test to which two-way ANOVA was applied. Tukey and Tukey–Kramer posthoc tests were applied when the sample sizes were same and different, respectively. p values less than 0.05 were considered as statistically significant.

## Supplementary Information

Supplementary Information 1.

Supplementary Information 2.

Supplementary Information 3.

## Data Availability

All data generated or analyzed during this study are included in this published article.

## Abbreviations

polyA: Polyalanine
polyL: Polyleucine
polyQ: Polyglutamine
polyS: Polyserine
SCA: Spinocerebellar ataxia
