# Supplementary material for: Conditioned medium from BV2 microglial cells having polyleucine specifically alters startle response in mice
Source: Sci Rep. 2022 Nov 4;12:18718. doi: 10.1038/s41598-022-23571-5 (PMC9636192; doi:10.1038/s41598-022-23571-5)
Supplement: Supplementary file 1 — Supplementary Information 1. [file 41598_2022_23571_MOESM1_ESM.pdf]

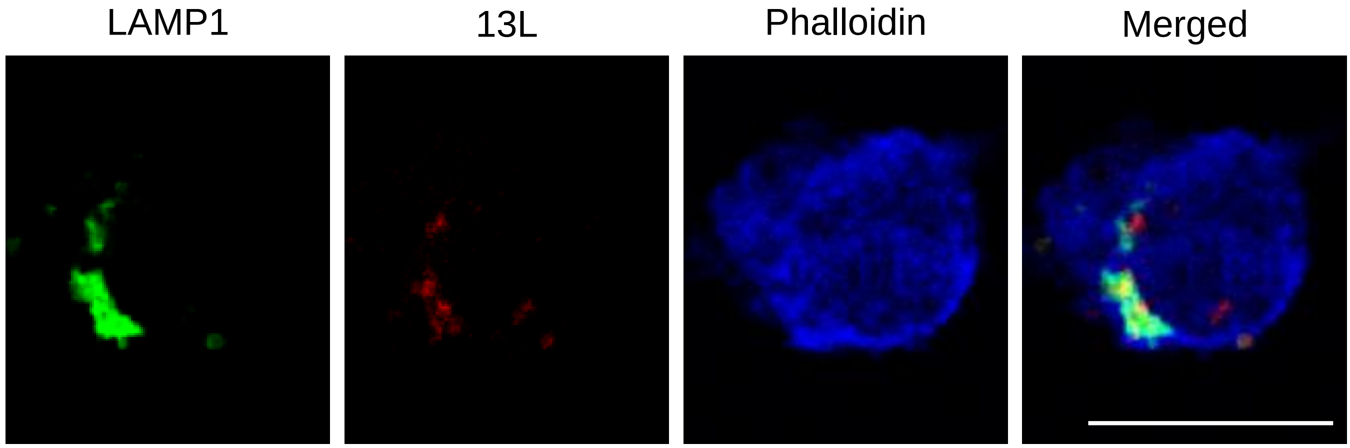

Supplementary Fig. S1. PolyL is delivered to lysosome.
Fluorescence staining of BV2 cells with 13L (red) with a lysosome marker LAMP1 (green) and phalloidin (blue).
