# Supplementary material for: Conditioned medium from BV2 microglial cells having polyleucine specifically alters startle response in mice
Source: Sci Rep. 2022 Nov 4;12:18718. doi: 10.1038/s41598-022-23571-5 (PMC9636192; doi:10.1038/s41598-022-23571-5)
Supplement: Supplementary file 2 — Supplementary Information 2. [file 41598_2022_23571_MOESM2_ESM.pdf]

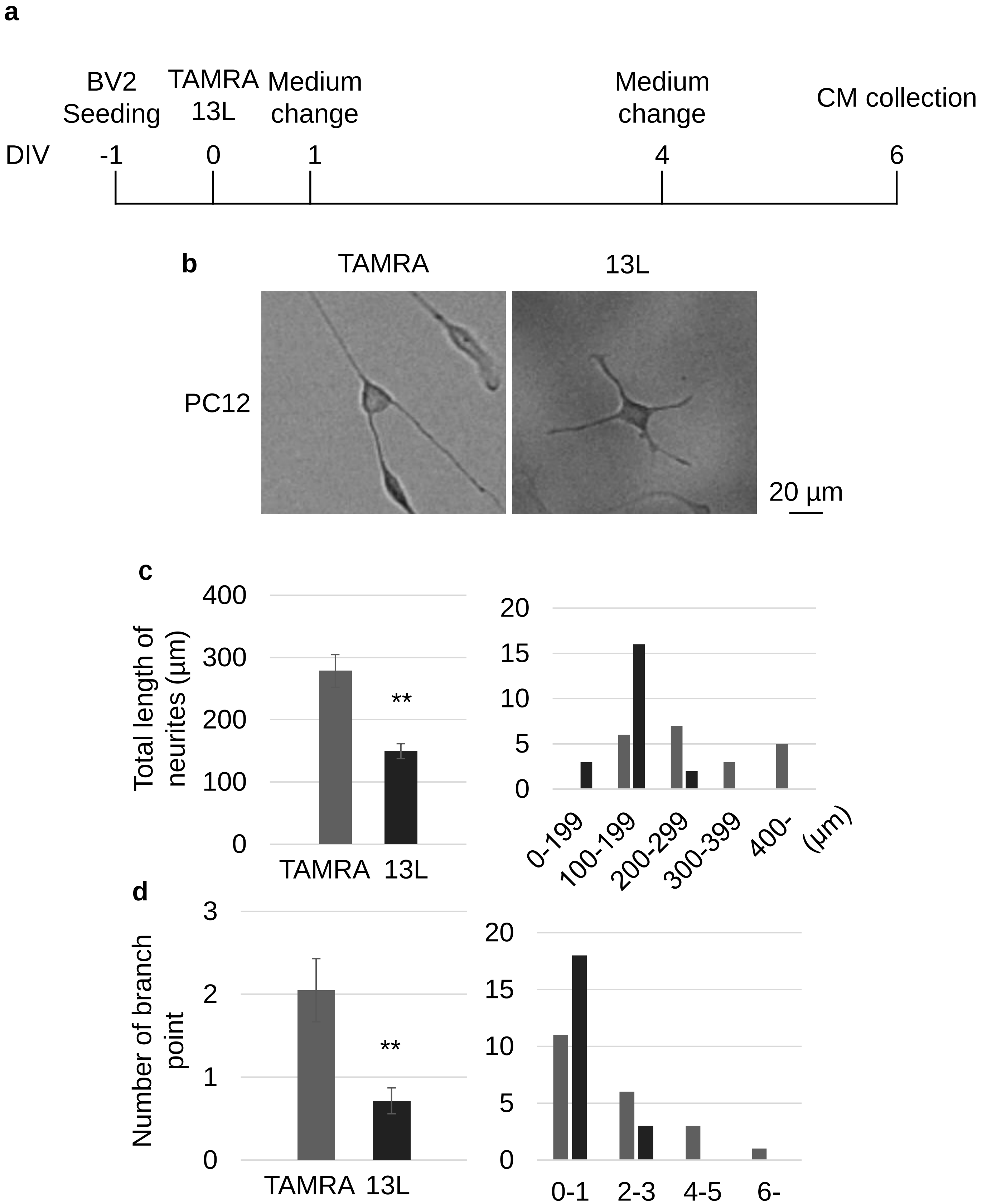

Supplementary Fig. S2. Morphological changes in differentiated PC12 cells given CM from 13L-treated BV2 cells at later time point.

(a) Time schedule of the experiment. (b-d) Quantification of the total length of neurites (c) and the number of branch point (d) of differentiated PC12 cells cultured with CM from TAMRA- or 13L-treated BV2 cells (n = 21 cells, each from 3 independent experiments). x-axis and y-axis of histograms indicate distribution of values and numbers, respectively. Representative images are shown in (b). Scale bar, 20 µm. Error bars represent SE. ANOVA, **p < 0.01.
