# Supplementary material for: Conditioned medium from BV2 microglial cells having polyleucine specifically alters startle response in mice
Source: Sci Rep. 2022 Nov 4;12:18718. doi: 10.1038/s41598-022-23571-5 (PMC9636192; doi:10.1038/s41598-022-23571-5)
Supplement: Supplementary file 3 — Supplementary Information 3. [file 41598_2022_23571_MOESM3_ESM.pdf]

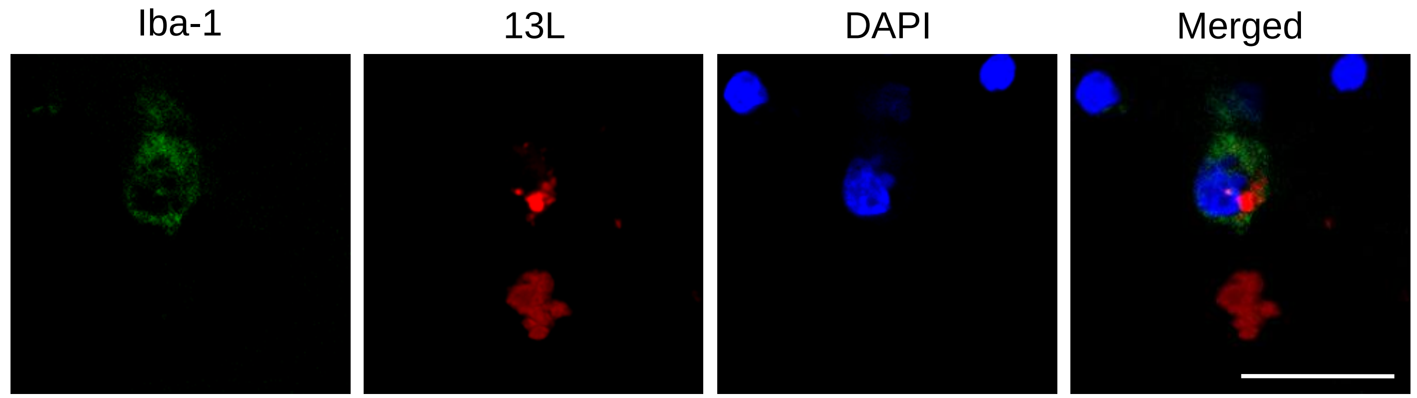

Supplementary Fig. S3. Recognition of polyL by microglia in vivo.
Immunostaining of the PnC with Iba 1 antibody (green). DAPI (blue) was also included in the staining. 13L (red) was injected in the PnC and the brain sections were collected 7 days later. Scale bar, 20 µm
